# Early surgeon experience with a fracture-specific humeral stem for proximal humerus fractures: a multicenter survey of predominantly reverse shoulder arthroplasty cases with an illustrative case

**DOI:** 10.1186/s12891-026-10239-8

**Published:** 2026-08-12

**Authors:** Matthias Regling, You Yang, Arthur de Gast, Gary F. Updegrove

**Affiliations:** 1 Stryker Trauma GmbH, Prof.-Küntscher-Str. 1, Schönkirchen, 24232 Germany; 2 Stryker Corporation, 10801 Nesbitt Avenue South, Bloomington, MN 55437 USA; 3 Stryker European Operations Ltd, Herikerbergweg 110, Amsterdam, 1101 CM Netherlands; 4 Department of Orthopaedics and Rehabilitation, Penn State Hershey Bone and Joint Institute, Penn State Milton S. Hershey Medical Center, 30 Hope Drive, Hershey, PA 17033 USA

**Keywords:** Shoulder arthroplasty, Total shoulder arthroplasty, Reverse shoulder arthroplasty, Hemiarthroplasty, Proximal humerus fracture, PHF

## Abstract

**Background:**

Shoulder arthroplasty has become an established treatment option for displaced and complex proximal humeral fractures, particularly in elderly patients. Fracture-specific stems are increasingly used in clinical practice; however, early data on surgeon experience with newly introduced convertible stem designs remain limited. This study aimed to assess orthopedic surgeons’ early intraoperative experience with a newly developed humeral fracture system (Tornier Perform Humeral System – Fracture, Tornier Inc., a wholly owned subsidiary of Stryker, Bloomington, MN, USA) using a structured survey as part of an early product surveillance program.

**Methods:**

A prospective observational survey was conducted among orthopedic surgeons in the United States and Canada who had performed surgery using the fracture stem. Case-level evaluations of intraoperative handling, technical features, and surgeon-reported comparisons with routinely used implant systems were collected. Descriptive statistics were used to summarize survey responses.

**Results:**

Twenty-six orthopedic surgeons submitted feedback on 47 surgical cases (45 primary cases, 2 revisions), including predominantly reverse configurations (46/47) and one hemiarthroplasty construct. All available stem fixation methods were used: cementless without interlocking screws (*n* = 24), cementless with interlocking screws (*n* = 14), and cemented (*n* = 9). Surgeons reported high levels of satisfaction and favorable ratings for ease of use. Across multiple comparative dimensions, the fracture system was frequently rated favorably compared with surgeons’ routinely used systems. Twenty-five of 26 participating surgeons indicated that they would consider using the system in future cases and would recommend it to others (missing data for one surgeon). Qualitative feedback described perceived benefits related to specific design features, including press-fit capability, bone graft window, proximal stem design, and the option for interlocking screw fixation.

**Conclusions:**

Surgeons reported favorable early intraoperative impressions of this fracture system. These findings provide exploratory insights into surgeon-reported usability and perceived design characteristics under early real-world use. Further studies are required to evaluate the clinical relevance of these surgeon-reported perceptions.

**Level of evidence:**

IV.

**Supplementary Information:**

The online version contains supplementary material available at 10.1186/s12891-026-10239-8.

## Background

Proximal humerus fractures (PHF) are common injuries in older adults and represent a growing surgical challenge as the population ages. These fractures frequently occur in the setting of osteoporosis and compromised metaphyseal bone quality, making surgical reconstruction difficult in displaced three- and four-part patterns [1, 2]. 

Treatment options range from nonoperative management to fixation or arthroplasty depending on fracture complexity, bone quality, and patient functional demand [3]. Reverse total shoulder arthroplasty (rTSA) has become an increasingly utilized option for complex PHF in elderly patients, as outcomes are less dependent on tuberosity healing than hemiarthroplasty [1, 4–6]. However, achieving stable humeral fixation in osteoporotic bone remains a major challenge, particularly when metaphyseal comminution limits implant fixation and tuberosity healing is uncertain [7, 8]. 

Successful fracture arthroplasty requires restoration of humeral height and version, secure implant fixation, and tuberosity repair, as tuberosity malposition or resorption is associated with inferior functional outcomes [8]. Modern fracture stems incorporate design features intended to improve metaphyseal fixation and facilitate tuberosity healing, including bone graft windows, suture fixation support, modularity, and optional interlocking screw fixation [7, 9, 10]. 

Despite these design advancements, limited data exist regarding early surgeon-reported intraoperative experience with recently introduced fracture systems in routine clinical practice.

The aim of this study was to characterize surgeons’ early intraoperative experience with a recently introduced fracture system based on structured survey responses collected during initial real-world use.

## Methods

After receiving standard market approval from respective authorities (U.S. Food and Drug Administration, Health Canada), a survey was conducted among U.S. and Canadian surgeons from July 2023 to December 2023. The survey questionnaire was specifically developed for this study and is provided as Supplementary File 1. The survey was designed as a prospective, single-arm observational study to collect voluntary surgeon feedback on technical and procedure-related aspects of the fracture stem (Tornier Perform Humeral System – Fracture, Tornier Inc., a wholly owned subsidiary of Stryker, Bloomington, MN, USA). The only applicable inclusion criterion was related to evidence of surgical use of the devices in scope of the survey; no specific exclusion criteria applied. In all cases, the devices were evaluated according to their intended use and approved indications.

Participation in the survey was voluntary. Surgeons were invited to provide feedback following clinical use of the system; however, the total number of eligible cases and response rates were not systematically tracked. Survey completion was not strictly enforced for individual cases, and it could not be verified whether all eligible cases were reported.

The survey questionnaire was developed internally by the study team based on clinical and procedural expertise with arthroplasty for PHF, and covered basic case details, an evaluation of the products and the related procedure, as well as a general feedback section. Questions included in the section on the evaluation of the product and procedure were limited to those aspects of shoulder arthroplasty that could be affected by the newly developed implants and instruments, i.e. questions related to glenoid preparation or fracture exposure, for example, were not asked. The survey included structured questions as well as open comment fields, addressing both perceived benefits and drawbacks, including areas for improvement and observed deficiencies. Further, certain questions presented to the surgeons were contingent upon the specific fixation technique employed.

The general feedback section was asked to be completed only once by each of the surveyed surgeons, i.e. on their first response; in addition, it was possible to update previously submitted ratings in the general feedback section with each subsequent response. In applicable cases, only the final response (reflecting the most recent subjective opinion and evaluation by the respective surgeon) was considered for the data analysis.

The questionnaire mainly applied bipolar 5- and 7-point Likert-type scale items with use of verbal labels for all points along the scales. All scale items offered an opt-out choice (‘no answer’), allowing participants to voluntarily bypass questions they did not want to answer. ‘No answer’ responses were retained as a distinct response category and included in the analysis, without imputation. Respondents could also provide open-ended comments, including their opinions on the fracture stem’s main benefits and drawbacks.

The web-based questionnaire was deployed using the Qualtrics survey platform, Version July 2023 (Qualtrics, Provo, UT, USA). Hard copy forms were offered and distributed via email. Coordination of the return of completed responses was facilitated by medical device sales representatives, who were formally trained in the technical and clinical application of the fracture stem, as well as the process of response return.

Open-ended answer fields related to subjective opinions on main benefits and drawbacks of the system were thematically coded. The questionnaire did not undergo formal psychometric validation, and responses reflect subjective surgeon-reported assessments. Analyses were limited to descriptive statistics, as the study was designed to characterize early intraoperative impressions rather than test predefined hypotheses. Quantitative data were summarized using measures of central tendency and distribution, and qualitative data were analyzed as relative frequencies. For the purpose of descriptive analysis, responses were grouped into categories reflecting overall evaluation. For 5-point scales, the two most favorable response options were considered “positive”, the middle category as “neutral”, and the two least favorable options as “negative”. For 7-point scales, the three most favorable response options were considered “positive”, the middle category as “neutral”, and the three least favorable options as “negative”. A descriptive subgroup analysis comparing responses from surgeons with and without consulting relationships was performed for key variables, including overall satisfaction, global ease-of-use evaluation, general comparison with routinely used systems, and likelihood of future system use.

Qualitative coding and data analysis were performed in a descriptive manner based on the survey responses; given the exploratory nature of the study, no independent validation or formal interobserver assessment was conducted.

Information on surgeon characteristics, including financial relationships with the manufacturer, was obtained retrospectively based on publicly available information and internal records following completion of data collection.

**Fig. 1:**
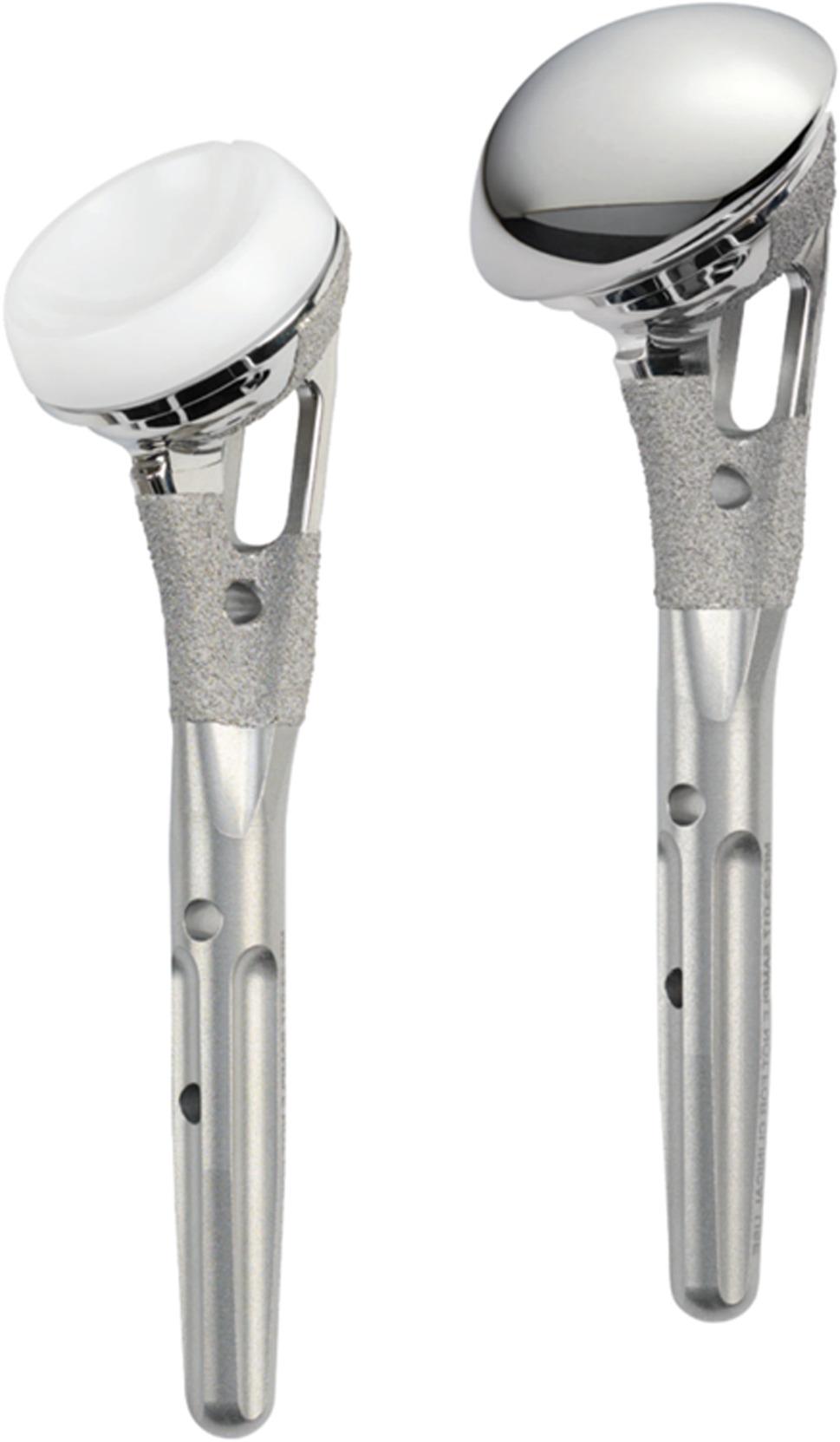
Representative fracture stems of the Tornier Perform Humeral System. Representative illustrations of the fracture stems shown in the reverse configuration (left) and in the anatomic configuration (right)

During the survey, the length offering for fracture stems was limited to 130 mm, with stem diameters of 7–17 mm increasing by 1 mm increments. The stem features a proximal bone graft window, a medial suture groove, and ridges on the sides of the metaphyseal bowl to help limit the movement of tuberosities. Additional features include a porous titanium coating, a neck-shaft angle of 140° as well as a low-profile metaphysis to the inlay proximal interface (Fig. 1).

The patient for the clinical case report was selected as a representative case from the cohort of patients who had been treated to date with the fracture stem at a tertiary care teaching center by an experienced board-certified orthopaedic surgeon. The patient was treated with the system following a standard surgical technique and was followed according to the regular standard of care. Written informed consent for publication of anonymized clinical images and accompanying case details was obtained from the patient.

## Results

### Demographics

In total, 26 surgeons, 25 from the USA and 1 from Canada, were included in the survey. The number of cases contributed per surgeon ranged from 1 to 5, with most surgeons contributing one or two cases. Most of the participating surgeons had many years of experience: the median time since completing their residency training program was 10 years (range: 3 to 38 years). Among the 26 participating surgeons, 12 had consulting relationships with the manufacturer (including one design surgeon) and contributed 24 cases, while 14 surgeons without such relationships contributed 23 cases.

4-part PHF were most frequent, followed by 3-part and 2-part fractures. The fracture stem was predominantly used in primary procedures and was almost exclusively implanted in rTSA constructs. Cementless fixation without interlocking screws was the most commonly applied fixation method (Table 1).

**Table 1:** Details of surgical procedure performed

PHF treated (*N* = 47)	*n* (%)
2-part	8 (17)
3-part	12 (26)
4-part	25 (53)
Missing data	2 (4)
Procedure (*N* = 47)	*n* (%)
Primary	45 (96)
Revision	2 (4)
Construct (*N* = 47)	*n* (%)
aTSA	0 (0%)
rTSA	46 (98%)
Hemiarthroplasty	1 (2%)
Stem fixation method (*N* = 47)	*n* (%)
Cemented	9 (19)
Cementless without interlocking screws	24 (51)
Cementless with interlocking screws	14 (30)

*aTSA* anatomic total shoulder arthroplasty, *rTSA* reverse total shoulder arthroplasty, *PHF* proximal humerus fracture

Sum of percentages may not equal 100% because of rounding

Information on the final implant configurations, including the stem sizes used and the details of the implanted reversed insert in rTSA procedures, are listed in Table 2.

**Table 2:** Details of final implant configuration: stem size and reversed insert

Stem size / diameter (*N* = 47)	*n* (%)
7 S	1 (2)
8 S	5 (11)
9	7 (15)
9 S	4 (9)
10	10 (21)
11	2 (4)
12	6 (13)
13	5 (11)
14	2 (4)
15	2 (4)
16	1 (2)
17	2 (4)
Reversed insert (*N* = 46)	*n* (%)
Diameter	
33 mm	0 (0)
36 mm	36 (78)
39 mm	6 (13)
42 mm	3 (7)
Missing data	1 (2)
Thickness	
+ 0 mm	28 (61)
+ 3 mm	14 (30)
+ 6 mm	3 (7)
Missing data	1 (2)
Angle	
Symmetric	39 (85)
10° Angled	6 (13)
Missing data	1 (2)
Constraint	
Standard	28 (61)
Retentive	16 (35)
Missing data	2 (4)
Option	
Conventional	34 (74)
Vitamin E	8 (17)
Missing data	4 (9)
Spacer use	
Yes	2 (4)
No	40 (87)
Missing data	4 (9)

The “S” designation for the stem size refers to a smaller collar diameter in comparison to the standard stems (34 mm vs. 38 mm)

Sum of percentages may not equal 100% because of rounding

In most cases (39/47, 83%), the implanted stem size corresponded to that of the final rasp, which is also used as a stem trial. In six cases (13%), a different stem size was selected: in 4 cases an undersized stem (-1 size) was used, and in 2 cases an oversized stem (+ 1 size) was implanted. In the remaining 2 cases (4%), information on the final rasp size was missing, and the difference between the rasp and the implanted stem size could not be determined.

In the single case of a hemiarthroplasty procedure, the fracture stem was implanted in combination with a centered head coupler and a 39 mm x 14 mm humeral head.

Information on the duration of the procedure was reported for 46 of the 47 cases; the median duration was 1 h and 37 min (range: 45 min to 3 h and 32 min).

### Evaluation of the product and procedure

**Fig. 2:**
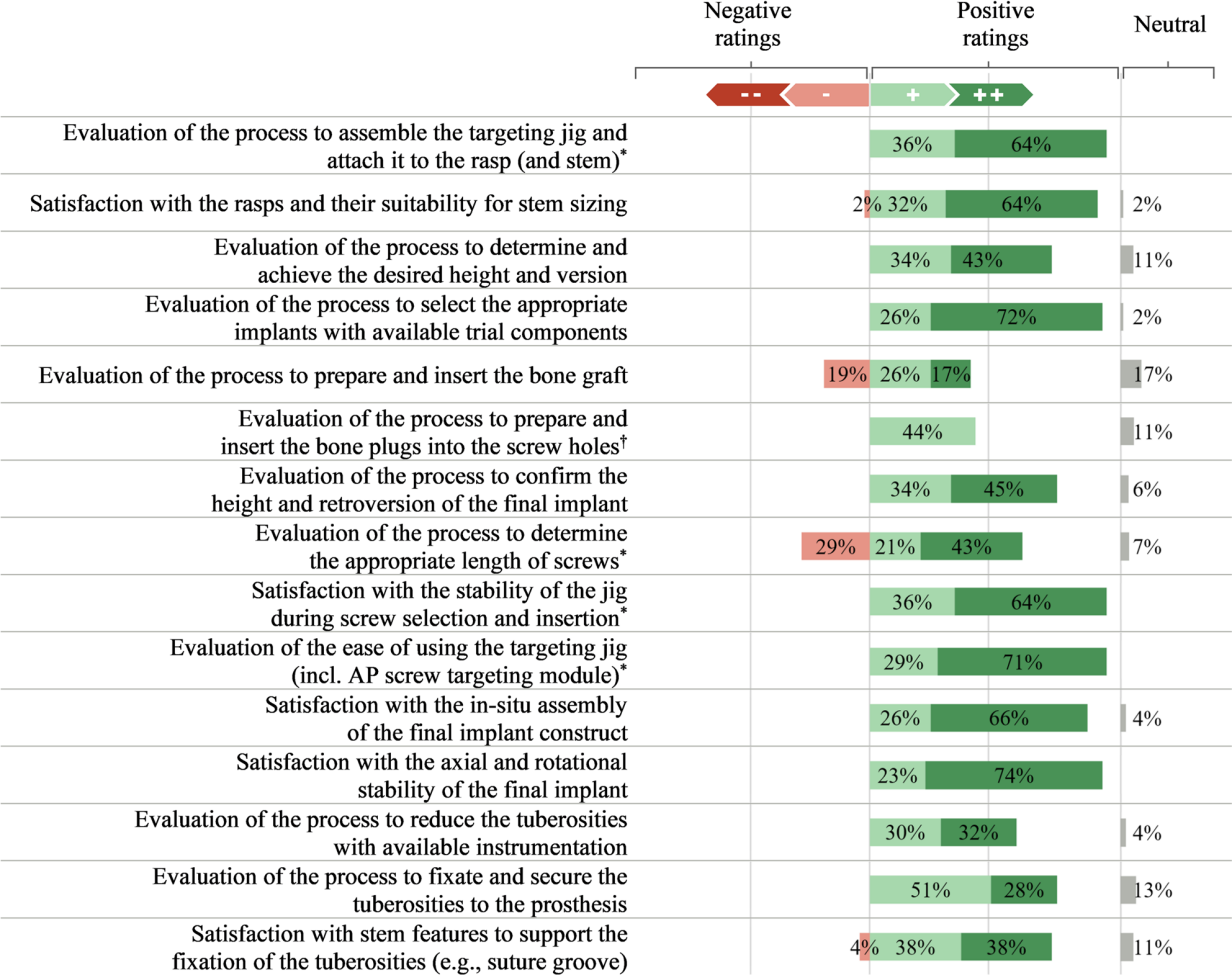
Surgeon responses to product and procedure evaluation items. Distribution of surgeon responses to Likert-type scale items evaluating the Tornier Perform Humeral System – Fracture and the related surgical procedure. Percentages are based on all reported cases (*N* = 47). Items marked with * were only applicable in cases using cementless fixation with interlocking screws (*n* = 14), and items marked with † were only applicable in cases using cemented stem fixation (*n* = 9). Values not displayed correspond to ‘no answer’ or missing responses and account for any difference from 100% of responses

Questionnaire items related to the evaluation of the product and procedure covered a range of intraoperative steps, including instrument handling, implant sizing, and fixation-related aspects. Overall, these items received predominantly favorable ratings, with most responses falling within the positive categories (Fig. 2).

Across the evaluated items, fundamental procedural steps such as stem sizing with rasps, adjustment of height and version, trialing for appropriate implants, as well as the in-situ assembly of the final implant construct and axial and rotational stability, were generally rated favorably, with positive responses observed in the majority of applicable cases.

Some aspects, however, showed greater variability in ratings. The process of preparing and inserting the bone graft using the dedicated instrumentation received mixed feedback, including ‘counterintuitive’ ratings in 9 of 47 cases (19%). Surgeons’ comments indicated that some surgeons encountered initial challenges with specific steps of the technique, including graft sizing, cutting through subchondral bone, and positioning of the graft. Similarly, the process of determining the appropriate screw length when using interlocking screw fixation demonstrated a broader distribution of responses, including ‘counterintuitive’ ratings in 4 of the 14 applicable cases (29%). Corresponding comments highlighted that some surgeons had concerns related to the concordance of measurement instruments and the usability of the depth gauge.

For some evaluation items, a proportion of cases was not assessed or rated by the surgeons. This was most notable for the process of reducing the tuberosities, which was not evaluated in 16 of 47 cases (34%). This was also observed for the process of preparing and inserting bone plugs into the screw holes, which was only applicable in cases using cemented stem fixation and was not evaluated in 4 of 9 applicable cases (44%), albeit within a small subset of cases. Smaller proportions of ‘no answer’ or missing responses were observed for other items, reflecting that not all aspects of the procedure were applicable or assessed in every case.

In addition to Likert-type evaluation items, the evaluation of the product and procedure included structured items related to the available range of implant sizes. The variety of stem sizes offered was considered ‘enough’ in 41 of 47 cases (87%), although some surgeons indicated that additional options would be desirable, including longer stems, larger diameters (> 17 mm), and expanded compatibility with smaller glenosphere diameters. In cases using cementless fixation with interlocking screws, the available range of screw lengths was considered ‘enough’ in all applicable cases.

**Fig. 3:**
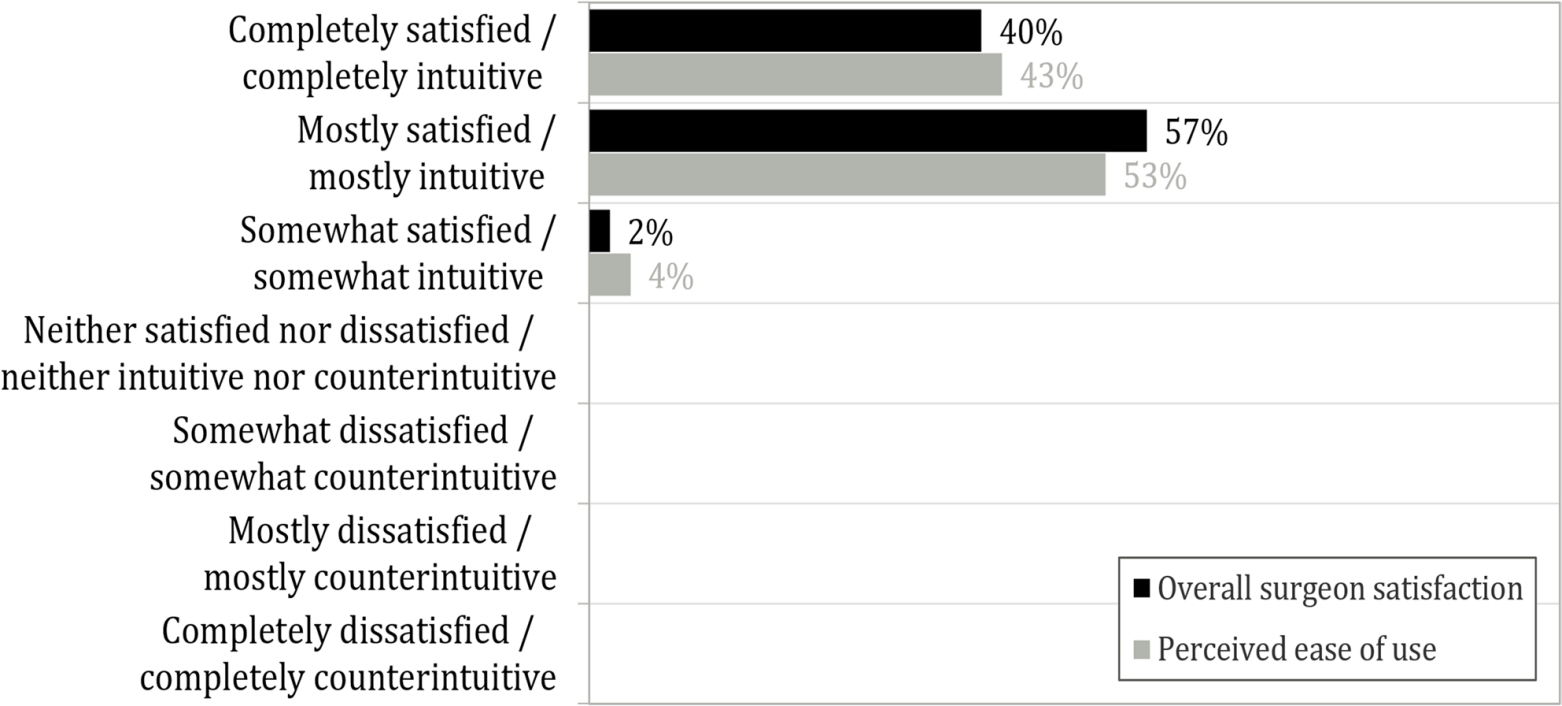
Overall surgeon satisfaction and perceived ease of use. Overall surgeon satisfaction with the Tornier Perform Humeral System – Fracture and general evaluation of its perceived ease of use. Percentages are based on all reported cases (*N* = 47)

Overall satisfaction and global ease-of-use ratings were uniformly positive, with all 47 cases rated within the three most favorable categories (‘completely’, ‘mostly’, or ‘somewhat’ satisfied/intuitive) (Fig. 3). A descriptive subgroup analysis showed similar patterns between surgeons with and without consulting relationships with the manufacturer. For overall satisfaction, top-2-box ratings (‘completely satisfied’ or ‘mostly satisfied’) were observed in 24/24 cases (100%) among consulting/design surgeons and 22/23 cases (96%) among non-consulting surgeons. The remaining non-consultant case was rated ‘somewhat satisfied’. Similarly, ease-of-use ratings were highly consistent between groups, with top-2-box ratings (‘completely intuitive’ or ‘mostly intuitive’) reported in 23/24 cases (96%) and 22/23 cases (96%), respectively. The remaining one case in each group was rated ‘somewhat intuitive’.

### General feedback

**Fig. 4:**
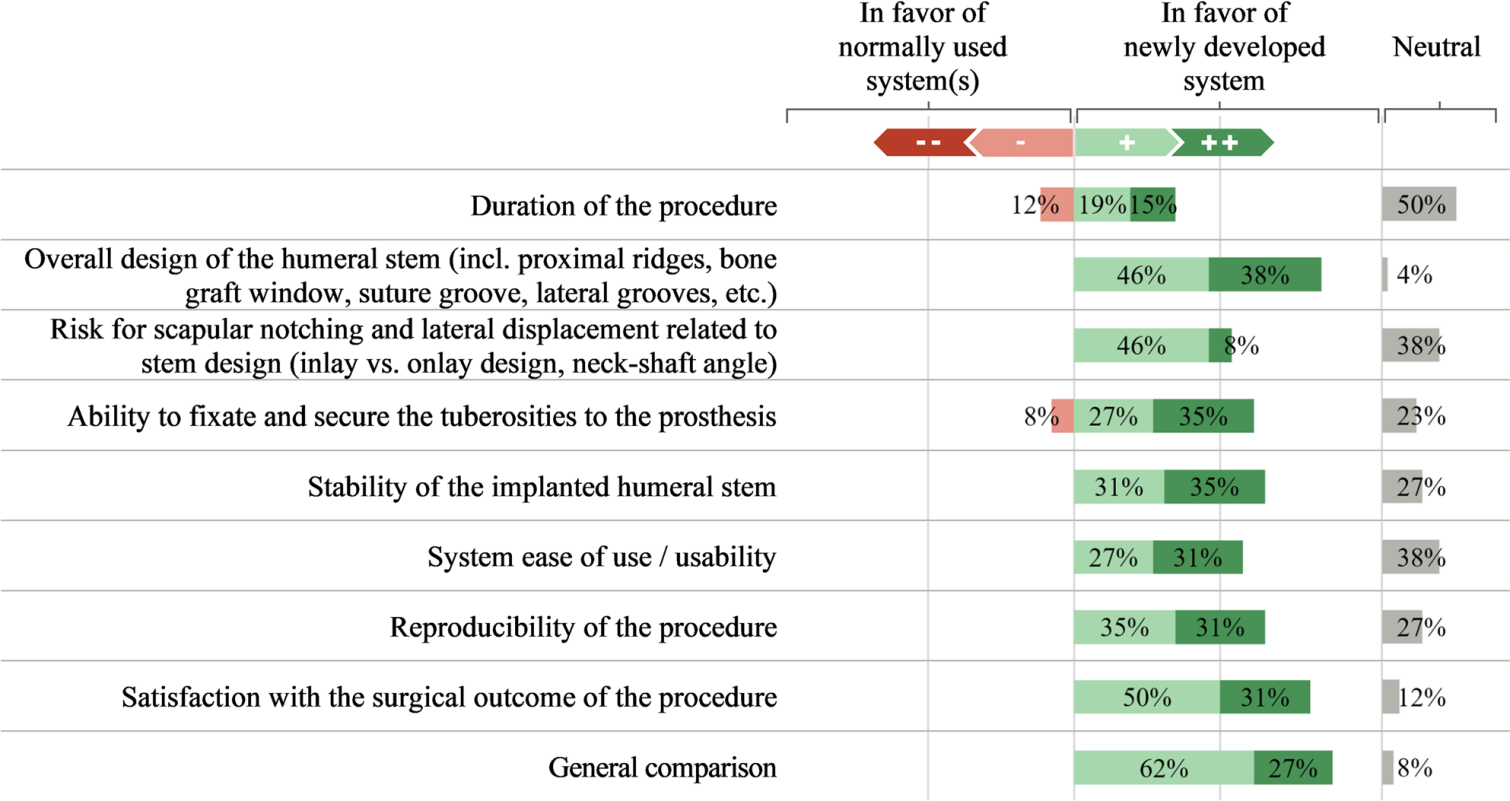
Comparative evaluation versus surgeons’ routinely used systems. Comparative assessment of the Tornier Perform Humeral System – Fracture relative to systems normally used by surveyed surgeons for the treated indication. Percentages are based on all participating surgeons (*N* = 26). Values not displayed correspond to ‘no answer’ or missing responses and account for any difference from 100% of responses

Information on normal system usage (multiple answers allowed) was provided by 23 of 26 surgeons (88%). Surgeons reported either exclusive use of a single system or concurrent use of multiple systems in their clinical practice. Most surgeons (15/23) indicated they normally use shoulder arthroplasty systems from Stryker, the manufacturer of the fracture stem. A total of 13 of 23 surgeons reported that they normally use shoulder arthroplasty systems from various other manufacturers, reflecting the diverse range of implant platforms routinely used in their practice. Comparative evaluation items of the fracture stem versus these other used systems included a wide array of different aspects, and surgeons rated these largely in favor of the fracture stem (Fig. 4). A descriptive subgroup analysis comparing surgeons with and without consulting relationships was performed for the general comparison between systems, which showed consistent patterns across surgeon-level assessments. For overall comparison with routinely used systems, median ratings were ‘better’ in both groups, with top-2-box responses (‘much better’ or ‘better’) reported by 10/12 surgeons (83%) among consulting/design surgeons and 13/14 (93%) among non-consulting surgeons. In both groups, the remaining responses included one ‘neither better nor worse’ rating, and one consulting/design surgeon had missing data. Finally, 25 of 26 surgeons (96%) confirmed they would use the system in the future and would recommend it to others; one surgeon had missing data. The descriptive subgroup analysis between surgeons with and without consulting relationships did not show materially different response patterns between groups, with median ratings of ‘very likely’ and all responses (except for one missing datapoint for one consulting surgeon) falling within the top two categories. Overall, these findings suggest consistent response patterns across surgeons with and without financial relationships with the manufacturer.

In addition, surgeons’ open-ended comments on their subjective opinions regarding main benefits were largely positive, with some drawbacks also noted (Table 3).

**Table 3:** Surgeons’ perception of main benefits and drawbacks of the Tornier Perform Humeral System – Fracture

Main benefits	*n* [# of mentions]
- Press-fit ability; avoidance of cement use and related adversities (e.g. time requirement and stress in the OR, risk of loosening), while keeping revisability high	13
- Bone graft window	9
- Ease of use / compatibility with familiar glenoid systems	8
- Variety of stem sizes with 1 mm increments, including small and large size options	8
- Stem design (140° angle, low profile/inlay design, smaller collar, metaphyseal bulk) and resulting fit and ease of fracture reduction	7
- Interlocking screw fixation option	5
- Rotational stability	3
- Tuberosity reduction and fixation	3
- Fracture graft clamp	2
- Proximal suture groove providing options	2
- Stability of fixation	2
- Variety of stem fixation options, cemented and cementless (with and without interlocking screws)	2
- Stability of rasps during trialing	1
- System dedication, fracture specific	1
- Targeting jig	1
Drawbacks	*n*
- Lack of proximal suture holes / suboptimal suture groove	9
- Limitations in length options / lack of long stems	3
- Rasping technique / stem size determination without use of canal reamers	3
- Cumbersome targeting jig	2
- Difficult fixation in fractures with compromised humeral calcar	1
- In-situ assembly of final implant construct	1
- Lack of Blueprint support	1
- Suboptimal screw retention on screwdriver tip	1

### Illustrative case: technique pearls from initial clinical experience

**Fig. 5:**
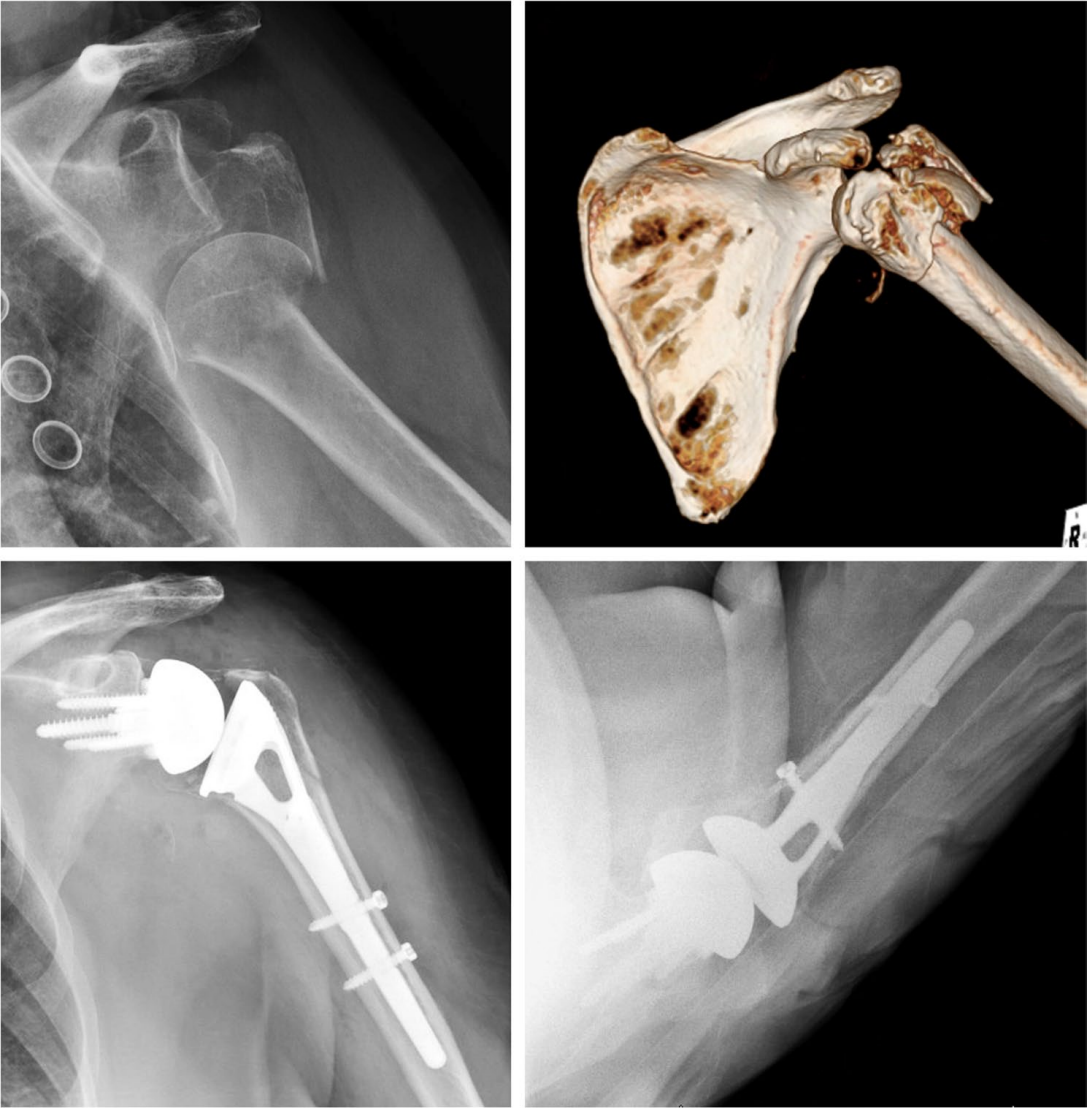
Pre- and post-operative imaging of an illustrative case. Pre- and post-operative imaging of the patient’s left shoulder. The top row shows pre-operative radiograph and computed tomography images demonstrating a proximal humerus fracture dislocation. The bottom row shows post-operative anteroposterior and axillary radiographs following treatment with reverse total shoulder arthroplasty using the Tornier Perform Humeral System – Fracture

A 78-year-old independent female with a BMI of 30 and a medical history of hypertension presented with a left shoulder fracture-dislocation following a fall from standing height. Imaging revealed a four-part PHF with comminution and displacement of both tuberosities (Fig. 5).

Closed reduction was performed in the emergency department, and a subsequent CT confirmed the complexity of the fracture pattern. Although she had no prior diagnosis of osteoporosis, clinical concern for poor bone quality supported the decision to proceed with rTSA.

Against this complex fracture scenario, the fracture stem described in this manuscript was selected because specific features of it were considered beneficial in this clinical setting for appropriate stability and facilitated tuberosity healing, i.e. the porous titanium coating for biologic fixation, the proximal bone graft window for structural autograft, suture grooves for tuberosity repair, and interlocking screws for enhanced rotational and axial stability. Also, the stem’s 1-mm size increments allowed for precise canal fit and version control in this case.

Surgery was performed under general anesthesia in the beach-chair position using a standard deltopectoral approach. The tuberosities were tagged with #5 braided sutures, and the humeral head was harvested for autograft. Glenoid preparation included placement of a + 3 mm lateralized baseplate with a central screw and four peripheral screws, followed by implantation of a 36 mm centered glenosphere.

The humeral canal was prepared with broaches up to size 10, with retroversion set at 20°. Humeral height was determined relative to the lateral cortex. Trial reduction with a 36 + 0 polyethylene liner confirmed satisfactory tension and stability. Two #5 sutures were passed through a small anterolateral canal drill hole to facilitate later vertical tuberosity repair.

The harvested humeral head was processed using the graft clamp, and after appropriate preparation inserted into the stem’s graft window. The final stem was inserted using the targeting jig with the planned height and version settings, achieving excellent press-fit comparable to the trial. An anterior-to-posterior interlocking screw was inserted first; this screw secured the construct in the set retroversion. Next, lateral-to-medial interlocking screws were placed percutaneously through the targeting jig, after first ensuring the deltoid wrapped anatomically around the humerus. Both drill sleeves were left in the jig during screw insertion to maintain stability. Once all screws were inserted, the targeting jig was removed, and the final polyethylene liner was impacted after verifying the chosen size and position.

Tuberosity repair was completed in accordance with techniques described by Boileau et al. [4], first by securing the greater tuberosity over the bone graft with two sutures around the stem. Then, the lesser tuberosity was secured with two additional sutures passed through the subscapularis and finally, vertical figure-of-eight sutures were tied through the canal to provide circumferential compression. After confirming reduction, soft tissue tension, and the absence of impingement, the lateral rotator interval between the anterior supraspinatus and superior subscapularis was closed. Intraoperative fluoroscopic imaging was used to confirm final component positioning, interlocking screw length, and tuberosity repair (Fig. 5). There were no intraoperative complications, and we achieved excellent tuberosity reduction and stable fixation of the humeral component.

The patient was discharged on postoperative day one with an abduction sling. At one-year follow-up, she demonstrated 150° of active forward elevation, 50° of external rotation, and radiographic evidence of tuberosity healing. She remains independently functional and continues osteoporosis management.

## Discussion

The early feedback from clinical users was largely encouraging, with positive ratings regarding overall surgeon satisfaction in all cases (Fig. 3). Among the most notable findings was the surgeons’ comparative evaluation against their normally used systems (Fig. 4). Most surgeons (23/26; 88%) rated the fracture system favorably in general, and the overall design of the humeral fracture stem was considered preferable over other systems by 22 of 26 surgeons (85%). Specific aspects that were rated in favor of the fracture stem included the ability to fixate and secure the tuberosities to the prosthesis and the stability of the implanted humeral stem. In the comparative assessment, these aspects were rated in favor of the fracture system by 16 of 26 surgeons (62%) and 17 of 26 surgeons (65%), respectively. Correspondingly, positive case-level ratings were observed in 36 of 47 cases (77%) for stem features supporting tuberosity fixation and in 46 of 47 cases (98%) for axial and rotational stability of the final implant. Adequate tuberosity fixation and implant stability have been shown to promote tuberosity healing, which is critical for maintaining reduction and avoiding complications such as suture pullout or resorption [4, 11]. Although the present survey did not examine postoperative healing or complication rates, the favorable surgeon ratings on these intraoperative aspects may be interpreted in light of this established clinical context. Within this framework, implant design features related to fixation and sizing may also play an important role in addressing intraoperative challenges. A recent review suggested that offering 1 mm diameter increments and optional interlocking screw fixation may support achieving appropriate implant fixation [5]. In the present survey, both of these features were highlighted by surgeons as perceived system benefits when asked for their subjective opinion (Table 3), aligning with the generally favorable ratings for implant stability observed in this study. Selected prior reports have highlighted challenges in achieving stable press-fit fixation in cases where the medullary canal size does not closely correspond to available implant sizes, including “in-between size” anatomical situations. Techniques such as matchstick autograft augmentation have been proposed to address this challenge [12]. In this context, finer size increments may theoretically reduce the likelihood of such scenarios, although this relationship has not been systematically evaluated. Overall, the preferences reported by surgeons in the present study may reflect the perceived value of additional flexibility in achieving implant fit and stability during intraoperative decision-making, rather than objective evidence of improved performance associated with specific size increments.

Beyond fixation and sizing, proper implant height is essential for restoring soft tissue tension and ensuring stable tuberosity fixation. Malpositioning, particularly excessive height or retroversion, has been associated with tuberosity migration, poor biomechanics, and inferior clinical outcomes [8]. Accurate restoration of humeral height, as emphasized by Boileau et al. [8] and Cagle et al. [13], is essential for optimizing tuberosity healing and improving postoperative function. In the present survey, items related to the implant height were rated positively in 37 of 47 cases (79%), although these ratings reflect intraoperative impressions and should be interpreted accordingly. Similarly, 14 of 26 surgeons (54%) perceived the risk of other prevalent post-operative complications potentially related to stem design, specifically scapular notching and lateral displacement, to be lower than with their normally used systems. According to a recent meta-analysis [14] of studies involving patients ≥ 65 years of age with complex PHF, scapular notching continues to occur in approximately 8–25% of cases, despite improvements in reported values from earlier studies. These data provide clinical context for such perceptions, while recognizing that postoperative outcomes were not evaluated in the present study.

Besides the size increments and optional interlocking screw fixation, several other key design considerations were highlighted by the surgeons as benefits, which included the bone graft window, the proximal stem design (among others, 140° neck shaft angle, low profile) and the resulting fit and ease of fracture reduction. These observations indicate which design features surgeons perceived as helpful for intraoperative handling and decision-making. Notably, many participating surgeons had extensive clinical experience in shoulder arthroplasty; while the findings remain subjective, they may provide insight into how experienced users perceive the system’s intraoperative handling and design characteristics during early clinical use. In addition, a descriptive subgroup analysis did not suggest materially different response patterns between surgeons with and without financial relationships with the manufacturer.

In contrast, and as detailed in previous sections, the survey revealed aspects that were not uniformly well received by responding surgeons. These points of critique likely reflect both device-specific considerations and the learning curve associated with early use of a newly introduced system, and they should be considered when interpreting the findings. They also highlight areas that may be considered in the ongoing refinement and evaluation of surgical techniques and implant design.

Several limitations should be considered when interpreting the results of this study. First, the distribution of reported cases was unbalanced, with the vast majority being primary procedures (96%) performed in a reverse shoulder arthroplasty configuration (98%). In addition, most cases were treated using cementless fixation without interlocking screws. As a result, the findings primarily reflect early experience with this configuration and may not be generalizable to other surgical scenarios, such as revision procedures, anatomic total shoulder arthroplasty, or cases requiring alternative fixation strategies. In addition, detailed patient-level characteristics (e.g., demographic and clinical factors such as age, bone quality, and fracture characteristics) were not collected, which may influence intraoperative decision-making and should be considered when interpreting the findings.

Second, the survey was based on voluntary participation, and the process by which surgeons were recruited as well as the total number of eligible participants were not systematically tracked. Consequently, response rates could not be determined. Similarly, completion of survey responses was not strictly enforced, and it could not be verified whether all eligible cases were reported. The potential for selection and reporting bias, including preferential reporting of favorable cases, should therefore be considered. Although the survey was designed to capture both positive and negative feedback, the potential for reporting bias cannot be excluded. Furthermore, multiple cases were contributed by individual surgeons, and clustering effects were not accounted for in the analysis.

Third, the study relied on a questionnaire specifically developed for this project that did not undergo formal psychometric validation. Survey responses were therefore based on subjective surgeon-reported assessments of intraoperative experience and may be influenced by individual expectations, prior experience, and familiarity with alternative systems.

Fourth, the survey focused exclusively on intra-operative impressions and did not include postoperative or patient-level clinical outcome data. As such, conclusions regarding implant performance, complication rates, or functional outcomes cannot be drawn from the present study.

Finally, the study was conducted within the framework of an industry-sponsored early product surveillance program, and financial relationships between some participating surgeons and the manufacturer were present (11 of 26 surgeons were consultants, and one was involved in the design of the system). These factors may have influenced aspects of participation or reporting and should be considered when interpreting the findings.

Taken together, these limitations highlight the exploratory nature of the present study and underscore the need for further independent studies with standardized data collection and clinical outcome assessment.

## Conclusions

This survey‑based study describes early intraoperative impressions of a convertible humeral fracture system collected during initial real‑world clinical use, primarily in rTSA cases. Orthopedic surgeons reported predominantly favorable evaluations across several aspects of system handling and design, with comparisons to their routinely used implants also generally trending positively. Overall assessments of usability and surgeon satisfaction were favorable. As these findings reflect early, surgeon‑reported intraoperative assessments, further prospective studies are required to evaluate their relevance in relation to clinical outcomes.

## Supplementary Information

Supplementary Material 1.

## Data Availability

The data that support the findings of this study are available from Stryker but restrictions apply to the availability of these data, which were used under license for the current study, and so are not publicly available. Data are however available from the corresponding author (Matthias Regling) upon reasonable request and with permission of Stryker.

## Abbreviations

aTSA: anatomic total shoulder arthroplasty
PHF: Proximal humerus fracture
rTSA: Reverse total shoulder arthroplasty

## References

[1] Christensen GV, O’Reilly OC, Bozoghlian MF, An Q, Nepola JV, Patterson BM. Trends in the treatment of proximal humerus fractures in the United States Medicare population. Seminars Arthroplasty: JSES. 2023;33(2):331–6.

[2] Klahs KJ, Hagen M, Scanaliato J, Hettrich C, Fitzpatrick KV, Parnes N. Geriatric proximal humerus fracture operative management: a Truven Health Analytics database study (2015–2020). J Shoulder Elb Surg. 2024;33(3):715–21. 10.1016/j.jse.2023.07.012 37573935

[3] Baker HP, Gutbrod J, Strelzow JA, Maassen NH, Shi L. Management of Proximal Humerus Fractures in Adults-A Scoping Review. J Clin Med. 2022;11:20. 10.3390/jcm11206140 PMC9604576 36294459

[4] Boileau P, Alta TD, Decroocq L, Sirveaux F, Clavert P, Favard L, et al. Reverse shoulder arthroplasty for acute fractures in the elderly: is it worth reattaching the tuberosities? J Shoulder Elb Surg. 2019;28(3):437–44. 10.1016/j.jse.2018.08.025 30573429

[5] Minarro JC, Sanchez-Sotelo J. Reverse Shoulder Arthroplasty for Proximal Humerus Fractures: A Review of Current Evidence. Curr Rev Musculoskelet Med. 2024;17(10):393–401. 39066981 10.1007/s12178-024-09919-6 PMC11371980

[6] Patel AH, Wilder JH, Ofa SA, Lee OC, Savoie FH 3rd, O’Brien MJ, et al. Trending a decade of proximal humerus fracture management in older adults. JSES Int. 2022;6(1):137–43. 35141688 10.1016/j.jseint.2021.08.006 PMC8811391

[7] Onggo JR, Nambiar M, Onggo JD, Hau R, Pennington R, Wang KK. Improved functional outcome and tuberosity healing in patients treated with fracture stems than nonfracture stems during shoulder arthroplasty for proximal humeral fracture: a meta-analysis and systematic review. J Shoulder Elb Surg. 2021;30(3):695–705. 10.1016/j.jse.2020.09.044 33157239

[8] Boileau P, Krishnan SG, Tinsi L, Walch G, Coste JS, Molé D. Tuberosity malposition and migration: Reasons for poor outcomes after hemiarthroplasty for displaced fractures of the proximal humerus. J Shoulder Elbow Surg. 2002;11(5):401–12. 12378157 10.1067/mse.2002.124527

[9] Imiolczyk JP, Moroder P, Scheibel M. Fracture-specific and conventional stem designs in reverse shoulder arthroplasty for acute proximal humerus fractures-A retrospective, observational study. J Clin Med. 2021;10(2):175. 10.3390/jcm10020175 PMC7825286 33419012

[10] Jo O, Borbas P, Grubhofer F, Ek ET, Pullen C, Treseder T, et al. Prosthesis designs and tuberosity fixation techniques in reverse total shoulder arthroplasty: influence on tuberosity healing in proximal humerus fractures. J Clin Med. 2021;10(18):4146. 10.3390/jcm10184146 PMC8468418 34575254

[11] Boyer E, Menu G, Loisel F, Saadnia R, Uhring J, Adam A, et al. Cementless and locked prosthesis for the treatment of 3-part and 4-part proximal humerus fractures: prospective clinical evaluation of hemi- and reverse arthroplasty. Eur J Orthop Surg Traumatol. 2017;27(3):301–8. 28238043 10.1007/s00590-017-1926-8

[12] Ouseph A, Lo EY, Montemaggi P, Krishnan SG. Cementless reverse shoulder arthroplasty technique to maximize press-fit fixation with humeral matchstick bone grafts. JBJS Essent Surg Tech. 2024;14(4):e23.00062. 10.2106/JBJS.ST.23.00062 PMC11444585 39364326

[13] Cagle PJ Jr., Reizner W, Parsons BO. A technique for humeral prosthesis placement in reverse total shoulder arthroplasty for fracture. Shoulder Elb. 2019;11(6):459–64. 10.1177/1758573218793904 PMC7094067 32269606

[14] Colasanti CA, Anil U, Rodriguez K, Levin JM, Leucht P, Simovitch RW, et al. Optimal combination of arthroplasty type, fixation method, and postoperative rehabilitation protocol for complex proximal humerus fractures in the elderly: a network meta-analysis. J Shoulder Elb Surg. 2024;33(10):e559–74. 10.1016/j.jse.2024.03.040 38734127

