# Supplementary material for: Early surgeon experience with a fracture-specific humeral stem for proximal humerus fractures: a multicenter survey of predominantly reverse shoulder arthroplasty cases with an illustrative case
Source: BMC Musculoskelet Disord. 2026 Aug 12;27:685. doi: 10.1186/s12891-026-10239-8 (PMC13471380; doi:10.1186/s12891-026-10239-8)
Supplement: Supplementary file 1 [file 12891_2026_10239_MOESM1_ESM.docx]

**Supplementary File 1. Survey Questionnaire**

The questionnaire was developed specifically for this study to capture surgeon experience with the Tornier Perform Humeral System – Fracture following standard clinical use. Questions were administered via an online survey platform, with case-specific items completed following each procedure and the General Feedback section completed once per participating surgeon. Skip logic and branching were applied where appropriate.

**SECTION 1: Case Details**

1. Surgeon *(free text)*
2. Hospital *(free text)*
3. City *(free text)*
4. Is this your first feedback submission for the Tornier Perform Humeral System – Fracture? *(Yes / No)*
5. Stryker sales representative *(free text)*
6. Date of surgery *(date field)*
7. Duration of procedure *(free text)*
8. Procedure type *(Primary / Revision)*
9. Construct *(Anatomic / Reverse / Hemiarthroplasty)*
10. Fracture type *(2-part / 3-part / 4-part)*
11. *Optional:* Upload anonymized radiographs or intraoperative images *(file upload)*

**SECTION 2: Evaluation of the Product and Procedure**

12. How did you fixate the final implant?
    *(Cemented / Cementless without screws / Cementless with screws)*
13. *(If cementless with screws)* Please evaluate the process to assemble the targeting jig and attach it to the rasp (and stem) *(5-point scale: Completely intuitive – Completely counterintuitive; includes “No answer” option)*
14. Please indicate the size of the last selected rasp *(free text)*
15. Please indicate your satisfaction with the rasps and their suitability for stem sizing
    *(5-point scale: Completely satisfied – Completely dissatisfied; includes “No answer” option)*
16. Please evaluate the process to determine and achieve the desired height and version
    *(5-point scale: Completely intuitive – Completely counterintuitive; includes “No answer” option)*
17. Please evaluate the process to select the appropriate implants with available trial components *(5-point scale: Completely intuitive – Completely counterintuitive; includes “No answer” option)*
18. *(If cementless with screws)* Did you utilize a 2.5 mm K-wire for stability while trialing? *(Yes / No)*
19. Please specify the details of the final implants selected for your patient: Stem size
    *(7s / 8s / 9s / 9 / 10 / 11 / 12 / 13 / 14 / 15 / 16 / 17)*
20. *(If anatomic or hemiarthroplasty construct)* Please specify the head configuration:

- Head coupler *(Centered / Low offset)*;
- Humeral head diameter [mm] / height [mm] *(free text)*

21. *(If reverse construct)* Please specify the reversed insert configuration:

- Diameter *(33 / 36 / 39 / 42 mm)*;
- Thickness *(+0 / +3 / +6 mm)*;
- Angle *(Symmetric / 10° angled)*;
- Constraint *(Standard / Retentive)*;
- Option *(Conventional / Vitamin E)*;
- Spacer used *(Yes / No)*

22. Please evaluate the variety of offered stem sizes *(Enough / Not enough – please specify)*
23. Please evaluate the process to prepare and insert the bone graft *(5-point scale: Completely intuitive – Completely counterintuitive; includes “No answer” and N/A option if not used)*
24. *(If cemented)* Please evaluate the process to prepare and insert the bone plugs into the screw holes *(5-point scale: Completely intuitive – Completely counterintuitive; includes “No answer” option)*
25. Please evaluate the process to confirm the height and retroversion of the final implant *(5-point scale: Completely intuitive – Completely counterintuitive; includes “No answer” option)*
26. *(If cementless with screws)* Please specify the lengths of the implanted Tornier Perform Fracture screws: Distal M/L screw, Proximal M/L screw, AP screw *(free text)*
27. *(If cementless with screws)* Please evaluate the variety of offered screw lengths
    *(Enough / Not enough – please specify)*
28. *(If cementless with screws)* Which technique(s) did you use for screw measurement? *(Drill bit / Depth gauge)*
29. *(If cementless with screws)* Please evaluate the process to determine the appropriate length of screws *(5-point scale: Completely intuitive – Completely counterintuitive; includes “No answer” option)*
30. *(If cementless with screws)* Please indicate your satisfaction with the stability of the jig during screw selection and insertion *(5-point scale: Completely satisfied – Completely dissatisfied; includes “No answer” option)*
31. *(If cementless with screws)* Please evaluate the ease of using the targeting jig (incl. AP screw targeting module) *(5-point scale: Completely intuitive – Completely counterintuitive; includes “No answer” option)*
32. Please indicate your satisfaction with the in-situ assembly of the final implant construct *(5-point scale: Completely satisfied – Completely dissatisfied; includes “No answer” option)*
33. Please indicate your satisfaction with the axial and rotational stability of the final implant *(5-point scale: Completely satisfied – Completely dissatisfied; includes “No answer” option)*
34. Please evaluate the process to reduce the tuberosities with available instrumentation
    *(5-point scale: Completely intuitive – Completely counterintuitive; includes “No answer” option)*
35. Which suture technique did you use to fixate the tuberosities? *(free text)*
36. Please evaluate the process to fixate and secure the tuberosities to the prosthesis
    *(5-point scale: Completely intuitive – Completely counterintuitive; includes “No answer” option)*
37. Please indicate your satisfaction with stem features to support the fixation of the tuberosities (e.g., suture groove) *(5-point scale: Completely satisfied – Completely dissatisfied; includes “No answer” option)*
38. Please indicate your overall satisfaction with the complete Tornier Perform Humeral System – Fracture *(7-point scale: Completely satisfied – Completely dissatisfied; includes “No answer” option)*
39. Please evaluate the ease of use / usability of the complete Tornier Perform Humeral System – Fracture *(7-point scale: Completely intuitive – Completely counterintuitive; includes “No answer” option)*
40. Did you identify any areas for improvement for the Tornier Perform Humeral System – Fracture? *(No / Yes – please specify)*
41. Did you observe any deficiencies and/or complications? *(No / Yes – please specify)*
42. Please share any additional comments you may have on the product and procedure
    *(free text)*

**SECTION 3: General Feedback**

43. In your opinion, what are the main benefits of the Tornier Perform Humeral System – Fracture? *(free text)*
44. In your opinion, are there any drawbacks of the Tornier Perform Humeral System – Fracture? *(free text)*
45. Which system(s) do you normally use for the treated indication? *(free text)*
46. Please evaluate the following aspects of the Tornier Perform Humeral System – Fracture in comparison to your normally used system(s):

- Duration of the procedure *(5-point scale: Much shorter – Much longer; includes “No answer” option)*;
- Overall design of the humeral stem (incl. proximal ridges, bone graft window, suture groove, lateral grooves, etc.) *(5-point scale: Much better – Much worse; includes “No answer” option)*;
- Risk for scapular notching and lateral displacement related to stem design (inlay vs. onlay design, neck-shaft angle) *(5-point scale: Much lower – Much higher; includes “No answer” option)*;
- Ability to fixate and secure the tuberosities *(5-point scale: Much better – Much worse; includes “No answer” option)*;
- Stability of the implanted humeral stem *(5-point scale: Much better – Much worse; includes “No answer” option)*;
- System ease of use / usability *(5-point scale: Much easier – Much more difficult; includes “No answer” option)*;
- Reproducibility of the procedure *(5-point scale: Much better – Much worse; includes “No answer” option)*;
- Satisfaction with the surgical outcome of the procedure *(5-point scale: Much more satisfied – Much less satisfied; includes “No answer” option)*

47. *If duration of procedure was rated “shorter” or “much shorter”:* Please specify the approximate time saved in your procedure *(free text)*
48. *If duration of procedure was rated “longer” or “much longer”:* Please specify the approximate time lost in your procedure *(free text)*
49. How does the Tornier Perform Humeral System – Fracture compare to the normally used system(s) in general? *(5-point scale: Much better – Much worse; includes “No answer” option)*
50. How likely would you be to use the Tornier Perform Humeral System – Fracture in the future? *(5-point scale: Very likely – Very unlikely; includes “No answer” option)*
51. How likely would you be to recommend the Tornier Perform Humeral System – Fracture to others? *(5-point scale: Very likely – Very unlikely; includes “No answer” option)*
52. Please share any additional comments you may have on the Tornier Perform Humeral System – Fracture in general *(free text)*
